# Editorial: Epigenomic polymorphisms: The drivers of diversity and heterogeneity

**DOI:** 10.3389/fgene.2022.1008178

**Published:** 2022-10-17

**Authors:** Tanvir-Ul-Hassan Dar, Naseem Akhter, Sajad Ahmad Dar

**Affiliations:** ^1^ Department of Biotechnology, Baba Ghulam Shah Badshah University, Rajouri, India; ^2^ Department of Neurology, Henry Ford Health System, Detroit, MI, United States; ^3^ Research and Scientific Studies Unit, College of Nursing, Jazan University, Jizan, Saudi Arabia

**Keywords:** epigenomic diversity, genomic variation, DNA methylation, phenotypic characters, epigenome

Evolutionary potential of a species is primarily driven by its genetic diversity, however accumulating evidence underscores the important role of epigenomic diversity (Agarwal et al., 2020; Neinavaie et al., 2021). Genomic diversity resulting from changes in DNA nucleotide sequences is not the only heritable information influencing population survival, evolution and ecology in plant and animal species; epigenomic variations, such as DNA methylation or chromatin states, percolate from generation to generation influencing phenotypic characteristics (Flatscher et al., 2012; Miryeganeh and Saze, 2019). Recent studies have found that epigenomic diversity substantially compensates for the loss of genomic variation(s) in small wild populations of genetically homogeneous colonies, thereby demonstrating an additional component of genomic variation(s) (Jueterbock et al., 2020; Mounger et al., 2021). This suggests that both genomic and epigenomic changes in plants and animals affect species and population diversity.

Even though research on inter-and intra-species heterogeneity has progressed significantly, the interplay between genomic (genetic) and epigenomic changes in the wild populations remains to be elucidated. The idea that epigenomic diversity can compensate for genomic diversity loss is relatively new and the extent and patterns of genomic and epigenomic diversity in eukaryotic species, especially those that are closely related but ecologically distinct, warrants comprehensive investigation.

In the present Research Topic, we have collated ten articles (eight original research and two reviews), illustrating the patterns of genomic and epigenomic diversity in eukaryotic species. Barrera-Redondo et al., in their review, have summarized the theoretical and technical bases for conducting domestication genomics, from acquiring a reference genome and genome assembly to population genomics, transcriptomics, epigenomics, and experimental validation of domestication-related genes. The mechanism of epigenetic changes and their dynamic role in maintaining genomic integrity during plant growth and reproduction have been reviewed here by Kumari et al. in an elaborated way.

In addition to genetic variation patterns linked to various environmental challenges (Hodgins et al., 2015; Neinavaie et al., 2021), mounting evidence suggests that epigenetic variation plays a role in ecology and this variation can be both environmentally induced and contribute to phenotypic plasticity (Ashe et al., 2021; Stajic and Jansen, 2021). In their research, Mounger et al. have dealt with differences between genetic and epigenetic parameters in *Spartina alterniflora* (*Sporobolus alterniflorus*, foundation plant)*,* across intertidal gradients. They used epigenotype-by-sequencing (epiGBS), in combination with environmental factors and plant phenotypic variation, in wild *S. alterniflora* populations to connect patterns of genomic and epigenomic diversity with environmental and phenotypic variations. A small but considerable amount of genetic and epigenetic diversity is accounted for by the habitat within populations. While differences in ABC transporter methylation patterns under various environmental conditions and their role in plant growth, development and response to biotic and abiotic stresses have been well documented (Tani et al., 2016; Moretti et al., 2017), little is known about variation in ABC transporter methylation patterns in native and non-native plant species. The results of changes in the methylation of ABC transporters of *Conyza canadensis* (*Erigeron canadensis*) in its native (North America) and non-native (Kashmir Himalayas) ranges are presented in this Research Topic by Shah et al. The DNA methylation of ABC transporter genes has been found to be lower in Kashmir Himalayas than in North America.

The B chromosome has recently been discovered to affect the cell’s DNA methylation status, thereby impacting the global gene expression profile (Mendioroz et al., 2015). In this Research Topic, Cardoso et al. have used immunocytogenetics to analyse the epigenetic DNA modification status of B chromosomes in cichlid fish (*Astatotilapia latifasciata*), and the effect of B chromosome presence on the global contents of 5-methylcytosine (5 mC) and 5-hydroxymethylcytosine (5hmC). They found that *A. latifasciata’s* B chromosome has an energetic pattern of DNA epimarks, and that its presence promotes the loss of 5 mC in females with the B chromosome’s gonads and the loss of 5hmC in males with the B element’s muscle.

FOXP3 (Forkhead box P3) is a member of the Forkhead/winged-helix family of transcription factors that causes X-linked autoimmune disorders in mice and humans (Schubert et al., 2001). In their Research Topic, Sadaf et al. have focussed on epigenetic changes and their link to the downregulated FOXP3 gene in female breast cancer patients. FOXO1 promoter methylation and expression at the mRNA and protein levels in different stages of breast cancer, as well as their relationship with various clinical indicators, are still to be investigated (Jiang et al., 2018; Xing et al., 2018). Using methylation-specific PCR, mRNA expression, and immunohistochemistry, Khan et al. have examined FOXO1 mRNA and protein expression in breast cancer. The downregulated protein expression and promoter hypermethylation of the FOXO1 gene are found to have a significant relationship.

Using whole-exome sequencing analysis, Alharazy et al. have explored genetic variations in genes related to vitamin D metabolism in Saudi Arabian families with vitamin D deficiency. Their study has revealed relevant and novel exonic missense variants in both DHCR7 and LRP2 genes, stressing the need to find their association with vitamin-D deficiency. Ochwedo et al. have assessed the genetic polymorphism and temporal stability of Pfs230 domain one and Pfs48/45 domain three in *Plasmodium falciparum* parasites from western Kenya. They found that the domains of the Pfs230 and Pfs48/45 from various malaria-prone regions, including areas where clinical trials are undertaken, should be followed indefinitely subsequent to the discovery of novel polymorphic sites. Srivastava et al. used an integrated transcriptomic method and bioinformatic analysis to uncover altered molecular processes that explain the underlying aetiology of Kawasaki disease (KD). Their approach revealed deregulated molecular mechanisms explaining the underlying etiology of KD which could aid in identifying therapeutic targets and a biomarker panel for early diagnosis and severity of this disease.

In summary, the Research Topic offers an updated assembly of articles assessing the genomic and epigenomic variations in health and diseases of different species of plants and animals. The recent development in methodologies and techniques, such as epigenotyping-by-sequencing, whole-exome sequencing, methylated DNA immunoprecipitation, chromatin immunoprecipitation assay, DNA epi-marks, immunocytogenetics and methylation-specific PCR will help researchers in investigating population genetics and trajectories of the evolution of mutations causing DNA methylation changes. This, combined with genome editing, could unravel the evolutionary significance of epigenome variations.
